# Task-related functional connectivity dynamics in a block-designed visual experiment

**DOI:** 10.3389/fnhum.2015.00543

**Published:** 2015-09-30

**Authors:** Xin Di, Zening Fu, Shing Chow Chan, Yeung Sam Hung, Bharat B. Biswal, Zhiguo Zhang

**Affiliations:** ^1^Department of Biomedical Engineering, New Jersey Institute of TechnologyNewark, NJ, USA; ^2^Department of Electrical and Electronic Engineering, The University of Hong KongHong Kong, Hong Kong; ^3^School of Chemical and Biomedical Engineering and School of Electrical and Electronic Engineering, Nanyang Technological UniversitySingapore, Singapore

**Keywords:** functional connectivity, dynamic connectivity, visual system, time-varying correlation coefficient, sliding window

## Abstract

Studying task modulations of brain connectivity using functional magnetic resonance imaging (fMRI) is critical to understand brain functions that support cognitive and affective processes. Existing methods such as psychophysiological interaction (PPI) and dynamic causal modeling (DCM) usually implicitly assume that the connectivity patterns are stable over a block-designed task with identical stimuli. However, this assumption lacks empirical verification on high-temporal resolution fMRI data with reliable data-driven analysis methods. The present study performed a detailed examination of dynamic changes of functional connectivity (FC) in a simple block-designed visual checkerboard experiment with a sub-second sampling rate (*TR* = 0.645 s) by estimating time-varying correlation coefficient (TVCC) between BOLD responses of different brain regions. We observed reliable task-related FC changes (i.e., FCs were transiently decreased after task onset and went back to the baseline afterward) among several visual regions of the bilateral middle occipital gyrus (MOG) and the bilateral fusiform gyrus (FuG). Importantly, only the FCs between higher visual regions (MOG) and lower visual regions (FuG) exhibited such dynamic patterns. The results suggested that simply assuming a sustained FC during a task block may be insufficient to capture distinct task-related FC changes. The investigation of FC dynamics in tasks could improve our understanding of condition shifts and the coordination between different activated brain regions.

## Introduction

Identifying brain connectivity, especially task-related connectivity, from functional magnetic resonance imaging (fMRI) is critical to understand how brain is organized to support cognitive and affective processes (Bullmore and Sporns, 2009, 2012; Friston, 2011; Passingham et al., 2013). The inference of task-related connectivity changes can be achieved by dividing data samples of different task periods into different conditions (after taking into account of hemodynamic delay) and then calculating connectivity metrics of each condition separately. This branch of methods has been widely applied to connectivity studies using structural equation modeling (Zhuang et al., 2005) and Granger causality (Wen et al., 2013). On the other hand, model-based methods, which generate models with or without task-related connectivity changes to test whether including additional parameters of task modulations can significantly improve model fit, have also gained popularity. This branch includes psychophysiological interaction (PPI), which is based on a simple regression model (Rao et al., 2008), and dynamic causal modeling (DCM), which is based on generative differential equation models (Friston et al., 2003). However, both branches of methods usually implicitly assume that the connectivity is sustained during a block-designed task consisting of identical stimuli.

The presumption of sustained connectivity during a block-designed task period may not be true. Evidence from high-temporal-resolution imaging techniques (such as electroencephalography [EEG] and magnetoencephalography [MEG]) has shown that stimuli or tasks could elicit rapid changes of functional connectivity (FC) or effective connectviity (Ploner et al., 2009; Hu et al., 2012; Zhang et al., 2012). In addition, accumulating evidence has shown that even fMRI FCs during the resting state are not sustained, but vary with time (Chang and Glover, 2010; Kang et al., 2011; Kiviniemi et al., 2011; Handwerker et al., 2012; Allen et al., 2014; Di and Biswal, 2015). However, task-related FC dynamics within a certain task period are seldom explored from fMRI data, which probably due to the low temporal resolution of fMRI (usually > 1 s). From a signal processing point of view, the low temporal resolution of fMRI and the resultant limited number of data samples make the estimation of connectivity dynamics in a short task period highly variable and less reliable. Hence, there is little knowledge so far about whether task-related FCs are sustained or transient in a task.

In the present study, we hypothesized that fMRI FCs in a simple block-designed visual checkerboard experiment are not sustained but change with time. To validate this hypothesis, we performed a rigorous analysis of FC dynamics in a block-designed visual experiment, where fMRI data were acquired with a relatively shorter repetition time (TR) of 0.645 s (Nooner et al., 2012) and could provide necessary high temporal resolution to unravel FC dynamics within task blocks. As compared with literature on dynamic FC, the novelty of this study is two-fold. Firstly, most of previous task-related FC studies were focused on difference of FC (1) during tasks and at rest, (2) in task periods with different stimulation level, or (3) in different cohorts of subjects. However, our study is aimed to investigate FC dynamics of normal subjects during a task with repeatedly presented identical stimuli. Secondly, the research on FC dynamics in the resting state has gained popularity in recent years, but few have investigated FC dynamics in a task. Our current study attempts to explore transient and rapid dynamics of FC in a simple visual task, which is largely different from previous investigation on resting-state data. Therefore, this study could add new information regarding the dynamic organization of the brain in a task. Specifically, we focused on FCs among visual regions which were consistently activated during visual stimulation and default mode network (DMN) regions which did not show consistent activations. The dynamics of FCs were studied in three steps: (1) a sliding window approach was used to estimate time-varying correlation coefficient (TVCC) at every time point before, during, and after the task; (2) the slope of TVCC at each time point was estimated to identify the temporal trend of TVCC; (3) correlation coefficient in four non-overlap experimental sub-periods were estimated and compared to validate the FC dynamics. In the second step, the slope of TVCC was examined because the slope of a line is an important parameter to describe both the direction and the steepness of the line. Therefore, the slope of TVCC at each sample can reflect along which direction the TVCC changes (i.e., increase, decrease, or keep unchanged) and how large the change is. In another word, the slope would provide a quantitative measure to gauge the change of TVCC. Our results showed that, FCs between higher visual regions and lower visual regions exhibited significant temporal evolution. However, such temporal evolution could not be observed from FCs between (1) bilateral activated regions, (2) inactivated regions, (3) activated regions, and inactivated regions. We also examined the dynamics of BOLD responses and found that BOLD responses in activated brain regions and FCs between activated brain regions showed different changing patterns.

## Materials and methods

### Task and fMRI data acquisition

We analyzed fMRI data from the enhanced Nathan Kline Institute (NKI)/Rockland sample (Nooner et al., 2012) of the international neuroimaging data-sharing initiative (INDI) (http://fcon_1000.projects.nitrc.org/indi/enhanced/). Institutional Review Board Approval was obtained for this project at the Nathan Kline Institute and at Montclair State University. Written informed consent was obtained for all study participants (Nooner et al., 2012). Only the visual checkerboard data with a TR of 645 ms and the MPRAGE (magnetization-prepared rapid acquisition with gradient echo) anatomical images were used in the current analysis. The short TR ensured that sufficient data samples in the task period (around 32 samples within a short block period of 20 s) can be exploited to investigate dynamics in FCs. Inclusion criteria for subjects in the enhanced NKI/Rockland sample were: (1) they did not have mental or physical disease that could affect brain functions, (2) their data had small head motions (<3 mm or 3°), and (3) their data do not violate standard fMRI safety criteria (Gountouna et al., 2010; Tomasi et al., 2014). In total 20 subjects (18–60 years, mean = 31.7 years) from this dataset were included in current study.

The fMRI data were recorded from a simple checkerboard visual experiment, where the checkerboard stimuli were presented in the center of the screen with a flickering frequency of 4 Hz. With a block design, the scan started with a 20 s rest condition (fixation) and followed by a 20 s checkerboard condition with three repetitions. After the third checkerboard block, there was an additional 35 s rest condition. The total scan time was about 2 m 35 s with totally 240 images acquired. The fMRI data were scanned using a multiband echo planar imaging (EPI) sequence with the following parameters: *TR* = 645 ms; *TE* = 30 ms; flip angle = 60°; voxel size = 3 mm^3^ isotropic; number of slices = 40. The MPRAGE images were scanned using the following parameters: *TR* = 1900 ms; *TE* = 2.52 ms; flip angle = 9°; voxel size = 1 mm^3^ isotropic.

### fMRI pre-processing and analysis

Functional MRI data were preprocessed using SPM8 (http://www.fil.ion.ucl.ac.uk/spm/) under MATLAB7.6 environment (http://www.mathworks.com/). Before processing, the first 14 functional images (around 9 s) were discarded. We have not performed slice timing since this dataset was scanned using a multiband imaging technique, in which multiple slices were excited simultaneously. The remaining images were motion corrected for each subject, and co-registered to the subject's high resolution anatomical image. The anatomical image was segmented using the new segment routine in SPM8. Then, the deformation field obtained from the segmentation step was applied to all the functional images to normalize them into standard MNI space (Montreal Neurological Institute). All functional images were smoothed using a Gaussian kernel (FWHM = 8 mm). Finally, the functional images were temporally filtered by a second order Butterworth lowpass filter with a cutoff frequency of 0.75 Hz. To quantify the amount of head motion, we calculated mean frame-wise displacement (FD) in the translational and rotational directions for each subject (Yan et al., 2013). The mean FD values in translation and rotation across subjects were 0.06 mm and 0.03°, respectively, while the maximum mean FD values in translation and rotation across subjects were 0.11 mm and 0.06°, respectively.

**Figure 1:**
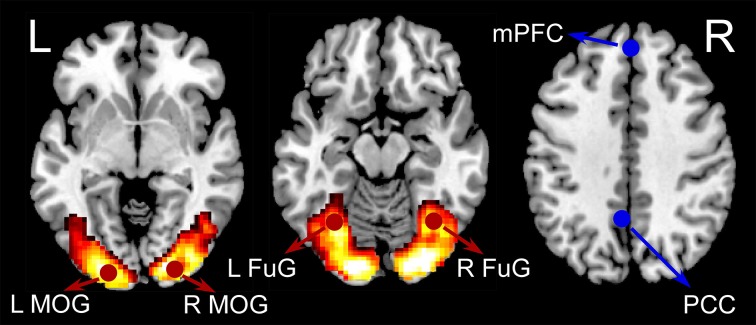
**The group-level BOLD activation map for visual checkerboard stimuli (hot color coded)**. Six ROIs used in the current analyses were displayed in red (activated) or blue (inactivated). The four activated ROIs were left middle occipital gyrus (LMOG) [MNI coordinates: right middle occipital gyrus (RMOG) (24, −91, 1), left fusiform gyrus (LFuG) (−33, −61, −14), and right fusiform gyrus (RFuG) (36, −58, −11). The two inactivated ROIs were medial prefrontal cortex (mPFC) (0, 51, 32) and posterior cingulate cortex (PCC) (−5, −49, 40)].

General linear model (GLM) was used to estimate activations relating to checkerboard presentations. A box-car function representing the checkerboard presentation block was convolved with the canonical hemodynamic response function (HRF). Other regressors in the GLM included six rigid body head motion parameters, their first order temporal derivatives, the first five eigenvariates from the signals in the white matter, and the first five eigenvariates from the signals in the cerebrospinal fluid (CSF). A default high-pass filter with a cutoff frequency of 1/128 Hz was incorporated in the GLM model. The beta maps representing activations of the visual stimuli were obtained by GLM from all subjects and then were entered into a second level analysis to assess consistent activations across subjects by using a one-sample *t*-test model. Four activated regions of interest (ROIs) were defined based on our previous study of an independent data set (Fu et al., 2014) to minimize selection bias (Kriegeskorte et al., 2009). The four activated ROIs were left middle occipital gyrus (LMOG) (MNI coordinates: −21, −91, −5), right middle occipital gyrus (RMOG) (24, −91, 1), left fusiform gyrus (LFuG) (−33, −61, −14), and right fusiform gyrus (RFuG) (36, −58, −11). Among the four visual ROIs, left MOG, and right MOG are lower visual areas while left FuG and right FuG are higher visual areas. Two inactivated ROIs in DMN: medial prefrontal cortex (mPFC) (0, 51, 32) (Dosenbach et al., 2010) and posterior cingulate cortex (PCC) (−5, −49, 40) (Jang et al., 2011) were also defined for comparison. The definitions of overall six ROIs were shown in Figure 1. ROIs were defined as 8 mm spheres centered at the MNI coordinates. The BOLD response of each ROI was the average of all voxels in this ROI. The first eigenvariate time-series data from the ROIs were extracted after adjusting for head motion parameters, signals from the white matter and CSF, and low-frequency drifts.

### Analysis of connectivity dynamics

#### Point-wise time-varying FC estimation

We used a sliding window TVCC estimation method to investigate the dynamics of FCs among different ROIs over the whole scan. The sliding-window TVCC estimation method may be the most fundamental and most widely-used method to infer the dynamic patterns of FCs (Hutchison et al., 2013a). The box-car task design function convolved with the canonical HRF was first regressed out to remove its impact on FC estimation (Whitfield-Gabrieli and Nieto-Castanon, 2012). Correlation coefficient between two ROIs was calculated at each time point using data samples within a Gaussian window $K_{h}\left(u\right)=\exp{\left(-u^{2}∕h\right)}_{\left\{|u|\leq h∕2\right\}}$, where *h* is the specified window size of 24 samples (16 s). This window size is shorter than the task block, so that important details of FC dynamics during the task period would not be overlooked. Also, since the fMRI data we used have a short TR of 645 ms, a window of 16 s includes around 24 samples in estimating the correlation coefficient. In literature, a window covering 24 samples is sufficient for estimating dynamic FC. For example, a window of 15 samples was used in Hutchison et al. (2013b) and a window of 16 samples was used in Handwerker et al. (2012). Other window sizes (14 and 18 s) were also used to calculate TVCCs and the results can be found in the Supplementary Materials.

#### Detection of changes in BOLD and FC

To investigate the changes of BOLD responses and FCs introduced by the task stimulation, we first examined whether BOLD or FC at each time sample after the stimulus onset was significantly different from the baseline values within the pre-stimulus period (−10 to 0 s). TVCCs were Fisher's z-transformed before the following analyses. For each subject, BOLD responses or FCs were averaged across three block cycles, and the mean of each BOLD or FC within the pre-stimulus period (−10 to 0 s) was the baseline value of this subject. Two-tailed one sample *t*-test was applied to check whether BOLD or FC at each time point after the stimulus onset was larger or smaller than their baselines at the group level. The significance threshold was corrected by the false discovery rate (FDR) procedure to address the problem of multiple comparisons (Benjamini and Hochberg, 1995).

#### Trend analysis of BOLD and FC

Further, we estimated the temporal trends of BOLD responses and FCs at each time point. For each subject, BOLD responses and FCs were averaged across three block cycles and then the slopes of each time point were estimated by a least-squares linear fitting of the samples within a rectangular window of 16 s. Two-tailed *t*-test was applied to check whether slope at each time point was significantly larger than zero (indicating an increasing trend) or smaller than zero (indicating a decreasing trend). The significance threshold was corrected by the FDR procedure to address the problem of multiple comparisons (Benjamini and Hochberg, 1995). The resulting time-varying slopes denoted whether BOLD or FC at each time point increased (larger than 0), decreased (smaller than 0), or sustained (not significantly different from 0).

#### FC in different experimental sub-periods

The sliding window method had unavoidable (and maybe large) estimation bias, because FCs or slopes of BOLD and FCs at one time sample were estimated from adjacent data samples, which may not have the same properties as the data at this time sample (Zhang et al., 2011). For example, when the window size for FC estimation is 16 s, the FC estimates in the range of 0–15 s after the stimulus onset were estimated from some samples before the stimulus onset. To further validate the FC dynamics estimated from the sliding window method, we examined the FCs within four non-overlapping sub-periods: (1) the pre-stimulation period (PRE-STIM: −10 to 0 s), (2) the early period of stimulation (EARLY-STIM: 0–10 s), (3) the late period of stimulation (LATE-STIM: 10–20 s), and (4) the post-stimulation period (POST-STIM: 20–30 s), where “0 s” denoted the onset time of one visual presentation block. FC in each sub-period was calculated as the correlation coefficients between data samples at two regions within one sub-period and averaged across three cycles after Fisher's z-transformed. A one-way repeated measure analysis of variance (ANOVA) was performed to examine whether there was any difference between FC in four experimental sub-periods (PRE-STIM, EARLY-STIM, LATE-STIM, and POST-STIM). Once ANOVA found significant difference (*p* < 0.05), *post-hoc* pairwise comparisons (paired *t*-test) were performed between two adjacent sub-periods to identify the temporal changes of FCs. The significance threshold was corrected by the FDR procedure to address the problem of multiple comparisons (Benjamini and Hochberg, 1995).

## Results

### BOLD responses and time-varying FC

**Figure 2:**
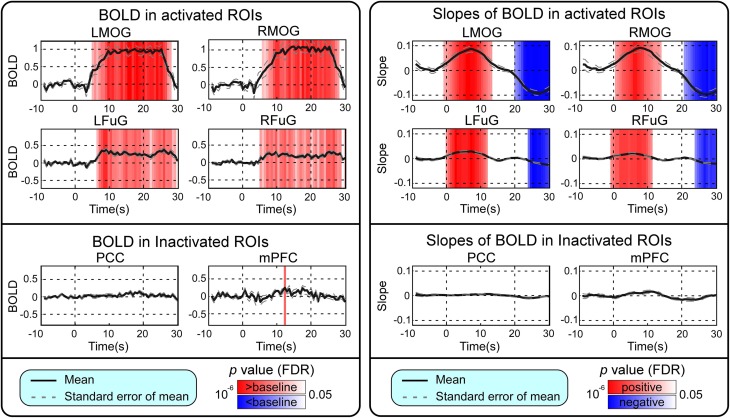
**Left:** Mean and standard error of mean (SEM) of BOLD responses in four activated ROIs and two inactivated ROIs. Two-tailed *t*-test was used to examine whether the BOLD after stimulus onset was larger or smaller than baseline at each time point. The time points with significantly larger or smaller (*p* < 0.05, FDR corrected) BOLD responses than baseline were highlighted with red or blue background, respectively. **Right**: Mean and SEM of slopes of BOLD in four activated ROIs and two inactivated ROIs. Two-tailed *t*-test was used to examine whether the slope of BOLD was larger than 0 or smaller than 0 at each time point. The time points with significantly positive or negative (*p* < 0.05, FDR corrected) slope values were highlighted with red or blue background, respectively. Stimulation period was from 0 to 20 s. Results were averaged across three blocks and all subjects.

**Figure 3:**
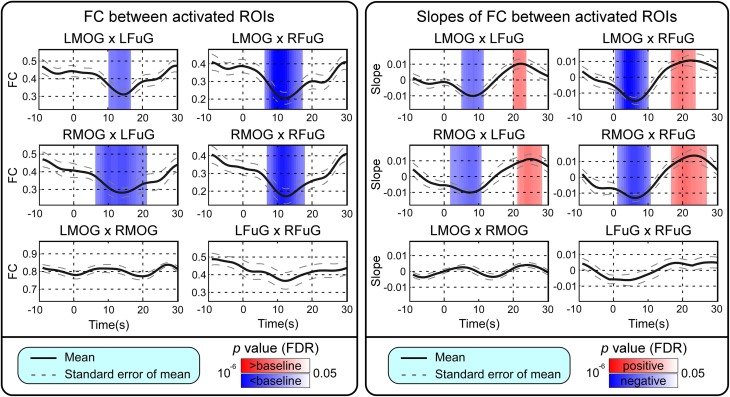
**Left:** Mean and standard error of mean (SEM) of FCs between four activated ROIs. Two-tailed *t*-test was used to examine whether the FC after stimulus onset was larger or smaller than that in baseline at each time point. The time points with significantly larger or smaller (*p* < 0.05, FDR corrected) FC than that in baseline were highlighted with red or blue background, respectively. **Right**: Mean and SEM of slopes of FCs between four activated ROIs. Two-tailed *t*-test was used to examine whether the slope of FC was larger than 0 or smaller than 0 at each time point. The time points with significantly positive or negative (*p* < 0.05, FDR corrected) slope values were highlighted with red or blue background, respectively. Stimulation period was from 0 to 20 s. Results were averaged across three blocks and all subjects.

**Figure 4:**
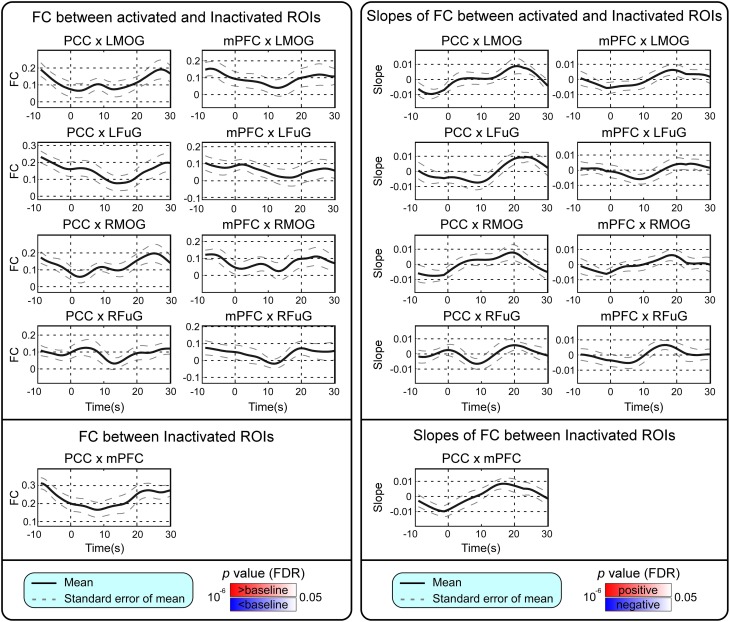
**Left:** Mean and standard error of mean (SEM) of FCs between the activated ROIs and inactivated ROIs, and FC between inactivated ROIs. Two-tailed *t*-test was used to examine whether the FC after stimulus onset was larger or smaller than that in baseline at each time point. The time points with significantly larger or smaller (*p* < 0.05, FDR corrected) FC than that in baseline were highlighted with red or blue background, respectively. **Right**: Mean and SEM of slopes of FCs between activated ROIs and inactivated ROIs, and slope of FC between inactivated ROIs. Two-tailed *t*-test was used to examine whether the slope of FC was larger than 0 or smaller than 0 at each time point. The time points with significantly positive or negative (*p* < 0.05, FDR corrected) slope values were highlighted with red or blue background, respectively. Stimulation period was from 0 to 20 s. Results were averaged across three blocks and all subjects.

BOLD responses and point-wise FC estimates, all of which were averaged across three block cycles, were shown in the left panels of Figures 2–**4**, respectively. In Figure 2, the BOLD responses of all activated ROIs (LMOG, RMOG, LFuG, RFuG) increased after stimulus onset (0 s) and showed significant larger activations than the baseline from 5 s after stimulus onset. These significant large BOLD activations lasted for around 20 s and went back to the baseline before 30 s. In contrast, BOLD responses of inactivated ROIs (PCC and mPFC) did not increase or decrease during the task. In Figure 3, FCs between higher visual ROIs (FuG) and lower visual ROIs (MOG) significantly decreased during the stimulation period (5–15 s). However, FC between two inactivated ROIs, FCs between one activated ROI, and one inactivated ROI (such as MOG and PCC), and FCs between bilateral ROIs (such as LMOG and RMOG, or LFuG and RFuG) did not change and were almost sustained during the task period, as shown in left panels of Figures 3, 4.

### Trend analysis of BOLD and time-varying FC

Point-wise trend analysis showed that the BOLD responses and FCs had largely different trends in the task period (0–20 s), and the results were displayed in the right panels of Figures 2–4, respectively. Firstly, it can be seen from Figure 2 that BOLD of activated ROIs increased significantly while the BOLD of inactivated ROIs did not show any dynamic trends during the stimulation period (0–20 s). Secondly, the FCs showed significantly different dynamic patterns between different ROIs. FCs between visual ROIs at different activation levels exhibited significantly negative slopes during the stimulation period. But, FC between inactivated ROIs, FCs between inactivated ROIs and activated ROIs, and FCs between bilateral ROIs did not have any increasing or decreasing trends during the task period. The same trend analyses were also conducted on TVCC estimates obtained with other window sizes (14 and 18 s). The results in Supplementary Materials showed that, although the window size could influence TVCC estimation, the decreasing trend of FCs over the whole task period could still be observed by using a window size of 14 or 18 s.

### FC in different experimental sub-periods

**Figure 5:**
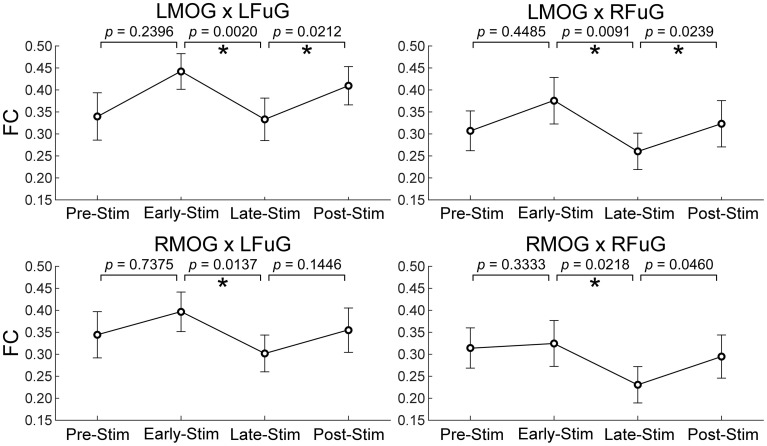
**Mean and standard error of mean (SEM) of FCs between higher and lower visual ROIs within four experimental sub-periods (the pre-stimulation period, PRE-STIM, −10 to 0 s; the early period of stimulation, EARLY-STIM, 0 to 10 s; the late period of stimulation, LATE-STIM, 10 to 20 s; the post-stimulation period, POST-STIM, 20 to 30 s.)** Here “0 s” denoted the onset time of one visual presentation block. The *p*-values of FC difference between adjacent sub-periods were presented, and significant difference (*p* < 0.05, FDR corrected) between FCs within adjacent sub-periods was marked using asterisks.

One-Way repeated measures ANOVA only identified significant FC difference among four sub-periods on those FCs between higher and lower visual ROIs (*p* = 0.0029 for LMOG × LFuG; *p* = 0.0213 for LMOG × RFuG; *p* = 0.0272 for RMOG × LFuG; *p* = 0.0342 for RMOG × RFuG). FCs with significant difference in different four experimental sub-periods (i.e., LMOG × LFuG, LMOG × RFuG, RMOG × LFuG, and RMOG × RFuG) were displayed in Figure 5. It can be seen that FCs between visual ROIs at different activation levels in the EARLY-STIM sub-period were significantly larger than that in the LATE-STIM sub-period. Moreover, it can be seen that there was a slight increase in FCs between PRE-STIM and EARLY-STIM sub-periods, but the significant level could not pass the FDR-corrected threshold. Similarly, there was also a slight increase in FCs in POST-STIM compared with that in LATE-STIM, although it could only pass the FDR-corrected significant threshold at LMOG × LFuG and LMOG × RFuG. The results in Figure 5 were consistent with the results shown in the left panel of Figure 3, where FC between activated ROIs was significantly decreased in the time range from 10 to 20 s. Therefore, the significant difference of FCs in different sub-periods further validated the FC dynamics in the task.

## Discussion

In the present study, we performed a detailed examination of the FC dynamics in a simple block-designed visual experiment, and found that FCs between the visual areas at different activation levels did not only vary between conditions (rest vs. task), but also changed within the task block (a significant decreasing trend during the task period). Our analysis revealed that (1) the FCs during the task period had different temporal dynamic behaviors; (2) FCs between spatially distributed visual regions could not persist at a high level but decreased within the task period. This was in contrast to BOLD responses in visual regions, which exhibited sustained activations over almost the whole task condition.

### Possible physiological basis of task-related FC dynamics

The transient connectivity changes between the visual areas were in line with neurophysiological evidence. At the neuronal level, neurons in visual areas showed increased synchronization within a second after stimulus onset (Bichot et al., 2005; Gregoriou et al., 2009). Similar phenomena have been observed in human using EEG (Cavanagh et al., 2009) and MEG (Gross et al., 2004). In addition, because the stimulation used in the present study was a very simple flickering checkerboard, the predictive value of the stimuli would decrease after brief presentation at the beginning of the block. Therefore, the communications between lower visual areas of the MOG and higher visual areas of the FuG may not be kept during the whole block period. Most interestingly, however, activated BOLD responses remained at a high level during the stimulation period, which was largely different from the transient changes of FCs. This may suggest a disassociation between FCs and BOLD responses. That is, both the lower and higher visual areas process information of the visual stimuli, while the communication between the lower and higher visual areas might be associated with the predictive values of the stimuli.

The physiological origin and mechanism of the observed task-related FC dynamics are difficult to be determined from the data and results in the present study. However, we speculate that the transient FC changes in the visual task might be caused by or related to (1) the predictive coding mechanism of the visual system, (2) the stimulus saliency, (3) the limited capacity of the brain for information integration, and (4) fluctuations of alpha band synchronizations.

First, the predictive coding mechanism might be one of the potential causes of the decrease on FCs between different visual sub-systems (Rao and Ballard, 1999; Brown et al., 2011; Bastos et al., 2012; Clark, 2013). Recently, there is increasing evidence to show that visual cortex is a multi-layered prediction system where the higher level sub-system could automatically adjusts its probabilistic representations so as to predict the inputs of the lower level sub-system (Summerfield et al., 2008; Kok et al., 2011; Bastos et al., 2012; Olsen et al., 2012). The connections between higher and lower visual sub-systems allow the prediction and error-correction cycles occur concurrently so that the higher visual sub-system could adjust itself to gradually minimize the prediction error. Once the higher visual sub-system successfully predicts the lower visual sub-system activities, no further action needs to ensue. The feedforward connection neurons are suppressed by feedback connection and thus finally show endstopping property. FCs between higher and lower visual sub-systems might be influenced by the connection neurons responsible for encoding and transferring the prediction errors. That is, these FCs increase when the connection neurons fire vigorously due to the large prediction error. In contrast, when the prediction error is small, connection neurons show little response so that the FCs decreases. That might explain why we could observe a slight increase in FCs between higher and lower visual sub-systems during the early stage of the block. When identical stimuli are presented repeatedly, the higher visual sub-system gradually adjusts its probabilistic representations to minimize the prediction error to a low level. As a result, the feedforward connection neurons would be suppressed, and FCs between higher and lower visual sub-systems during the late period of block would be decreased. On the other hand, the predictive coding mechanism might also lead to lower FCs during task blocks compared with those during rest periods. During the rest periods, the activity in the lower visual sub-systems is mainly resting state spontaneous activity, which might be susceptible to the random mental state or uncertain weak visual stimuli. The higher visual sub-systems can hardly find a certain probabilistic representation to the lower visual sub-systems and thus prediction error would maintain at some certain level. But during the visual task, since the activity in lower visual system is dominated by simple identical visual stimuli, the higher visual sub-system could gradually find an appropriate representation to minimize the prediction error. The feedforward connection neurons would show more endstopping property than that in the rest periods since the prediction error could be minimized to a smaller level. This might explain why lower FCs compared with those in the rest periods could be observed at the end of the task block.

Second, another possible cause of the task-related FC dynamics is the stimulus saliency, which refers to the ability of the stimulus to disrupt the current cognitive focus and elicit an attentional or behavioral switch (Downar et al., 2000). Activations of different brain regions are highly synchronized in response to the onset of new stimulation and give positive contribution to the original synchronization during the rest. Hence, FCs remain (slightly increase) at a high level after the onset of the visual stimulation block. Such task-related synchronization gradually decreases with the repetition of stimulation (at short and constant inter-stimulus interval) which would give negative contribution to the spontaneous synchronization, and, therefore, FCs fall down in the latter period of the stimulation block where the stimulation contained no salient information. Electrophysiological evidence has shown that stimulus repetition could significantly modulate brain responses and synchronization. For example, repetition of laser stimulation could decrease the magnitude of evoked brain potentials (Iannetti et al., 2008), and repetition of visual stimulation could lead to increased gamma-band synchronization within and between early and higher visual areas (Brunet et al., 2014). However, the current experimental design is insufficient to validate whether the FC dynamics are modulated by saliency or not, because the visual stimulation in this experiment was presented with the same intensity/pattern and at the same inter-stimulus interval and the only salient information was the stimulation onset. In order to test whether FCs are modulated by saliency of stimulation, we can deliver visual stimuli at varying and unpredictable inter-stimulus intervals and/or with different intensity/pattern in future experiments.

Third, FC dynamics may also be explained by the limited capacity of these activated brain regions for information integration and exchange (Marois and Ivanoff, 2005; Shanahan, 2012). The capacity of the activated brain regions is largely consumed by the first several stimuli and fewer resources are left to process subsequent stimuli. As a consequence, FCs could only maintain at a high level at the beginning of a block of stimulation and decrease afterwards.

Forth, FCs among visual ROIs generally were reduced during the checkerboard condition, as compared with the fixation condition. These are in line with several recent studies showing that FCs within the visual system were reduced during various tasks, as compared with those during resting state (Cole et al., 2014; Spadone et al., 2015). The BOLD signals of visual regions were highly synchronized in resting-state (Lowe et al., 1998; Biswal et al., 2010). And the high FCs were thought to associate with band-limited power fluctuations, specifically the alpha band power (He et al., 2008). Therefore, the reduced FCs during the checkerboard condition compared with the fixation condition may reflect reduction of alpha band synchronizations (Betti et al., 2013). This speculation (FC dynamics are correlated with alpha band synchronizations) could be validated by simultaneously acquired EEG-fMRI data in the visual checkerboard task.

### Methodological considerations

Correlation coefficient is a commonly used metric to assess FC between brain regions. When being used to infer time-varying FC, correlation can be estimated from short-windowed data segments, but the selection of window size for optimal estimation of time-varying FC is challenging. In this study we carefully selected appropriate window size in the sliding window approach to infer time-varying FC sample by sample. We also tested the estimation of TVCC using other fixed windows and the results were summarized in Section Explanation and Example for Regressing Out Task-related BOLD Responses of the Supplementary Materials. Overall, although the window size has a considerable influence on point-wise TVCC estimation, the decreasing trend of FCs over the whole task period could still be observed by using other fixed window sizes (if it was not too long or too short) in time-varying FC estimation.

It should also be noted that, regressing out task-related BOLD activities is a crucial pre-processing step in identifying task-related FC dynamics using sliding-window TVCC estimation. Correlation is a statistical metric that is only applicable for stochastic processes (spontaneous BOLD activity), but not for deterministic processes (task-activated BOLD activity). So, mathematically we need to remove task-related BOLD activities for correct estimation of TVCC. Importantly, if deterministic task-related BOLD activities were not removed before calculating TVCC, they would definitely cause dynamic changes of TVCC (please refer to Section Explanation and Example for Regressing Out Task-related BOLD Responses of the Supplementary Materials for a detailed explanation and an example). But, these dynamic TVCC patterns estimated from task-related BOLD activities are not physiologically relevant because they can be explained by well-studied task-related BOLD activities. Therefore, it is important to regress out task-related BOLD activities to make sure the correctness of the FC estimation method and to ascertain the physiological significance of observed FC dynamics. In the present study, the considerable task-related FC dynamics we observed were not estimated from task-related BOLD activities, so they can provide new information about the organization of the brain that cannot be provided by task-related BOLD activities.

Lastly, one possible limitation of our analysis is that, the FC dynamics of three block cycles are very large, because of the very low SNR of BOLD signal. So, we averaged the BOLD responses and FCs across three cycles before statistical comparisons across subjects and between time periods. The large variability in FC dynamics across cycles may be caused by noise, but it may also be physiologically relevant. In future, it will be interesting to study whether FC dynamics of separate cycles are stable or not from new experimental data, for example, with more cycles and more participants.

### Implications and future studies

The current task-related FC analyses suggested that simply dividing the samples in different task conditions and then calculating connectivity measures may not be sufficient to infer FC dynamics during a task period. We checked the sample-by-sample time-varying FC of the visual stimulation block and found significantly different changing patterns of FCs during the stimulation period. In addition, effective connectivity models that examine task related changes, such as PPI (Friston et al., 1997) and stochastic DCM (Friston et al., 2003), usually assume a constant FC over a condition. However, as implied by the current analyses, explicitly modeling the transient patterns of connectivity may be needed to advance our knowledge about how brain regions dynamically exchange information in a task period.

This study was based on a simple block-designed visual task in normal healthy people. We would like to pursue the following directions in future to further examine the generalizability and implications of the findings. A more complicated experiment should be designed to investigate how FC dynamics within the task period could be modulated by stimulation parameters (such as stimulus intensity and inter-stimulus interval) and psychological factors (such as sensory modality, saliency, and attention) for a more comprehensive study of the task-related FC dynamics and the underlying brain mechanisms. Second, we are interested to check whether such task-related FC dynamics are different in different cohorts of individuals (such as people with a specific neurological and psychiatric disease). Alterations in FCs have been associated with a wide range of neurological and psychiatric diseases (Greicius et al., 2003; Greicius, 2008; Fox and Greicius, 2010), but the clinical implications of task-related FC dynamics have been rarely explored. If task-related FC dynamics are found to be altered by neurological and psychiatric diseases, they may lead to a better understanding of the neuropathology and eventually, diagnostic, and prognostic indicators. Third, it will also be interesting to study the inter-subject difference of the task-related FC dynamics. Since the individual differences of FC have been attributed to age (Barber et al., 2013), sex (Kilpatrick et al., 2006), BOLD activation in task (Mennes et al., 2010), and even personality factors (Zhou et al., 2014), it is also possible that task-related FC dynamics could be linked to above individual factors. Therefore, more individual factors should be collected in future experiments to explore this possibility. If task-related FC dynamics are correlated with certain individual factors, they may be potentially used as biometric measures. For example, it was suggested in (Zilverstand et al., 2014) that dynamic FC correlated with task difficulty could be used as neurofeedback measures for individuals.

## Author contributions

XD, ZZ, and BB conceived and designed the study. ZF, SC, and YH analyzed the data. ZF, ZZ, and XD wrote the article. ZZ, XD, and BB made the critical revision of the article. All authors discussed the results and implications and commented on the manuscript at all stages.

### Conflict of interest statement

The authors declare that the research was conducted in the absence of any commercial or financial relationships that could be construed as a potential conflict of interest.

## Abbreviations

FC, FCs: functional connectivity
TVCC: time-varying correlation coefficient
MOG: middle occipital gyrus
FuG: fusiform gyrus.
