# Supplementary material for: Task-related functional connectivity dynamics in a block-designed visual experiment
Source: Front Hum Neurosci. 2015 Sep 30;9:543. doi: 10.3389/fnhum.2015.00543 (PMC4588125; doi:10.3389/fnhum.2015.00543)
Supplement: Supplementary file 1 [file Presentation1.PDF]

Supplementary Materials for

Task-related Functional Connectivity Dynamics in a Block-designed Visual Experiment

Xin Di[#], Zening Fu[#], Shing Chow Chan, Yeung Sam Hung, Bharat B Biswal[*], Zhiguo Zhang[*]

[#] **These authors contribute equally**
*** Corresponding authors:**
Dr. Zhiguo Zhang
Nanyang Technological University
School of Chemical and Biomedical Engineering and School of Electrical and Electronic Engineering
62 Nanyang Drive, North Spine, Block N1.3, B2-12, 637459, Singapore, Singapore
E-mail: zgzhang@ntu.edu.sg

Dr. Bharat B Biswal
New Jersey Institute of Technology, Newark, New Jersey, United States

Department of Biomedical Engineering

New Jersey Institute of Technology, University Heights Newark, 07102, New Jersey, USA

E-mail: bbiswal@yahoo.com

1. Trend Analysis of BOLD and Time-varying FC (using different window sizes)

In the main text, a Gaussian window with size of 16 s was used to calculate FC and the slopes were estimated using FC estimates within a rectangular window of the same window size (16 s). Here, we used other window sizes to investigate the dynamic patterns in FC. Symmetric windows with size 14 s and 18 s were chosen for comparison.

BOLD responses and point-wise time-varying correlation coefficient (TVCC) estimates (Gaussian window with window size = 14 s and 18 s), all of which were averaged across three block cycles, were shown in the left panels of Figures A1 ~ A6, respectively. Firstly, it could be seen that, relatively shorter window sizes would introduce lager variation in correlation estimates while larger ones would smooth out many transients. Nonetheless, similar significant changes on FCs could still be observed in the results from these window sizes, such as that FCs between ROIs at different activation levels (MOG & FuG) decreased significantly during the stimulation period (5s ~ 15s).

On the other hand, the current point-wise trend analysis could also obtain several consistent transient patterns in BOLD and FCs. Results were displayed in the right panels of Figures A1 ~ A6, respectively. Firstly, except for the BOLD of RFuG (results of 14 s), in which a short period of increasing trend had been detected around the stimulation offset, other dynamic trends of BOLD were similar to the results in main text. Secondly, we could still observe most of the consistent transient patterns in FC, such as the significant decreasing trends in FC between at different activated levels during the stimulation period. There was only a minor difference in current results compared with the results in main text: the increasing trends of FC between LMOG and LFuG, and between RMOG and LFuG after the stimulation offset could not be identified significantly in the results of 14 s window size. It is important to note that spurious information would be wrongly identified by inappropriate window sizes: (1) a too short window size, because of only a few samples used, has a large estimation variability, which implies spurious transient events may also be detected; (2) a too long window could decrease the variability of FC at a higher risk of (i) smoothing out meaningful transient patterns and (ii) inaccurate estimation of FC since including samples in different periods. Therefore, we only reported the results (decrease of FCs after stimulation onset) that can be consistently observed from results using different window sizes.

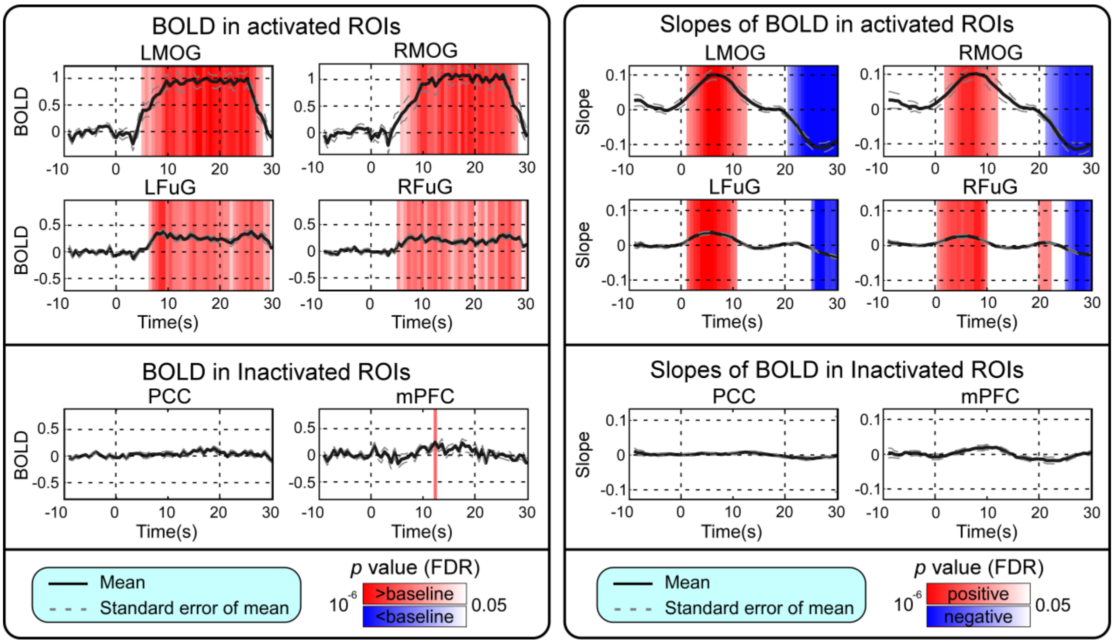

Figure A1 (*corresponding to Figure 2 in the main text, but with a different window size of 14s*). *Left*: Mean and standard error of mean (SEM) of BOLD responses in four activated ROIs and two inactivated ROIs. Two-tailed t-test was used to examine whether the BOLD after stimulus onset was larger or smaller than baseline at each time point. The time points with significantly larger or smaller ($p < 0.05$, FDR corrected) BOLD responses than baseline were highlighted with red or blue background, respectively. *Right*: Mean and SEM of slopes of BOLD in four activated ROIs and two inactivated ROIs. Two-tailed t-test was used to examine whether the slope of BOLD was larger than 0 or smaller than 0 at each time point. The time points with significantly positive or negative ($p < 0.05$, FDR corrected) slope values were highlighted with red or blue background, respectively. Stimulation period was from 0s to 20s. Results were averaged across three blocks and all subjects.

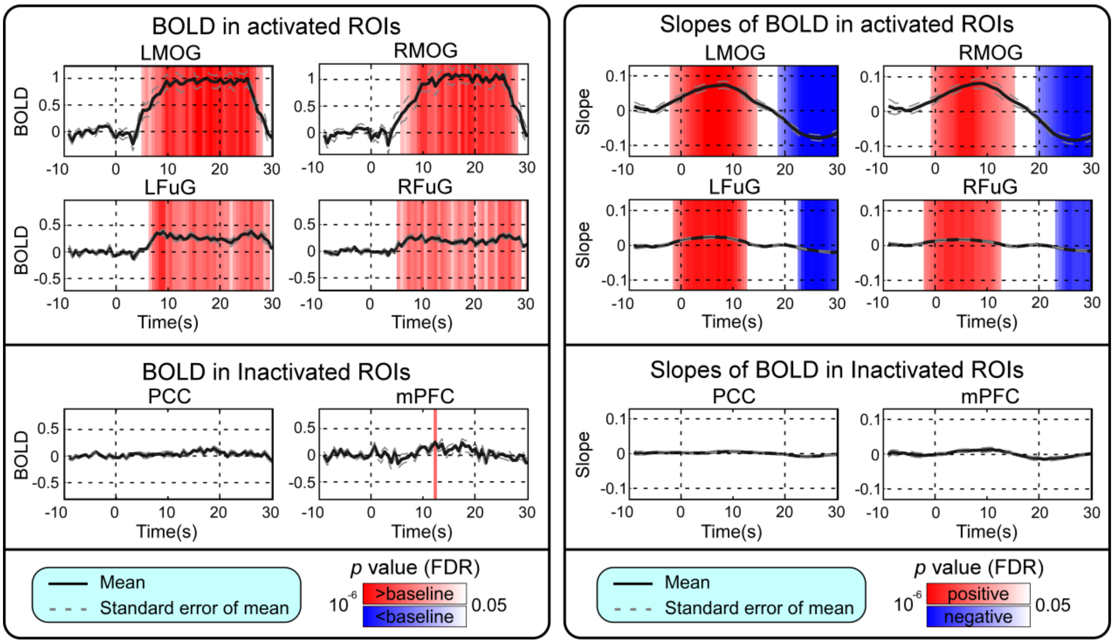

Figure A2 (*corresponding to Figure 2 in the main text, but with a different window size of 18s*). *Left*: Mean and standard error of mean (SEM) of BOLD responses in four activated ROIs and two inactivated ROIs. Two-tailed t-test was used to examine whether the BOLD after stimulus onset was larger or smaller than baseline at each time point. The time points with significantly larger or smaller ($p < 0.05$, FDR corrected) BOLD responses than baseline were highlighted with red or blue background, respectively. *Right*: Mean and SEM of slopes of BOLD in four activated ROIs and two inactivated ROIs. Two-tailed t-test was used to examine whether the slope of BOLD was larger than 0 or smaller than 0 at each time point. The time points with significantly positive or negative ($p < 0.05$, FDR corrected) slope values were highlighted with red or blue background, respectively. Stimulation period was from 0s to 20s. Results were averaged across three blocks and all subjects.

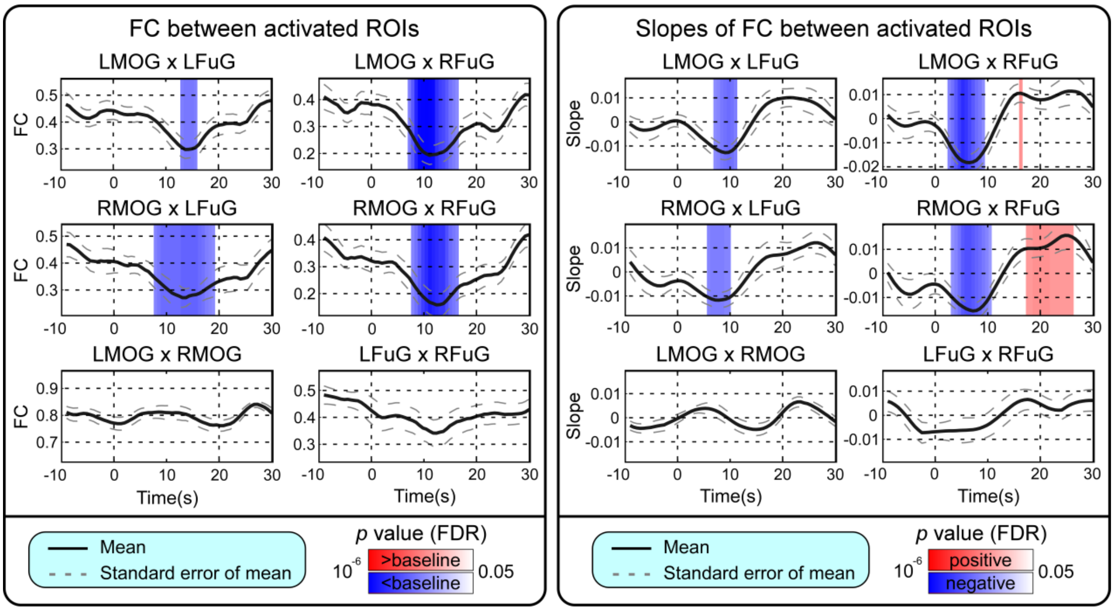

Figure A3 (*corresponding to Figure 3 in the main text, but with a different window size of 14s*). *Left*: Mean and standard error of mean (SEM) of FCs between four activated ROIs. Two-tailed t-test was used to examine whether the FC after stimulus onset was larger or smaller than that in baseline at each time point. The time points with significantly larger or smaller (p < 0.05, FDR corrected) FC than that in baseline were highlighted with red or blue background, respectively. *Right*: Mean and SEM of slopes of FCs between four activated ROIs. Two-tailed t-test was used to examine whether the slope of FC was larger than 0 or smaller than 0 at each time point. The time points with significantly positive or negative (p < 0.05, FDR corrected) slope values were highlighted with red or blue background, respectively. Stimulation period was from 0s to 20s. Results were averaged across three blocks and all subjects.

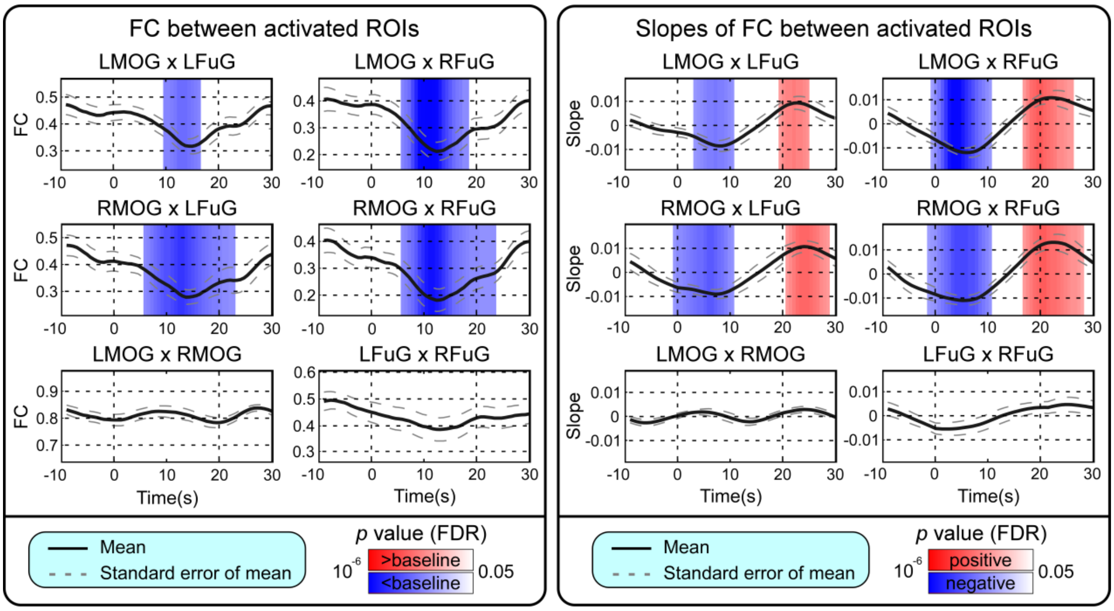

Figure A4 (*corresponding to Figure 3 in the main text, but with a different window size of 18s*). *Left*: Mean and standard error of mean (SEM) of FCs between four activated ROIs. Two-tailed t-test was used to examine whether the FC after stimulus onset was larger or smaller than that in baseline at each time point. The time points with significantly larger or smaller ($p < 0.05$, FDR corrected) FC than that in baseline were highlighted with red or blue background, respectively. *Right*: Mean and SEM of slopes of FCs between four activated ROIs. Two-tailed t-test was used to examine whether the slope of FC was larger than 0 or smaller than 0 at each time point. The time points with significantly positive or negative ($p < 0.05$, FDR corrected) slope values were highlighted with red or blue background, respectively. Stimulation period was from 0s to 20s. Results were averaged across three blocks and all subjects.

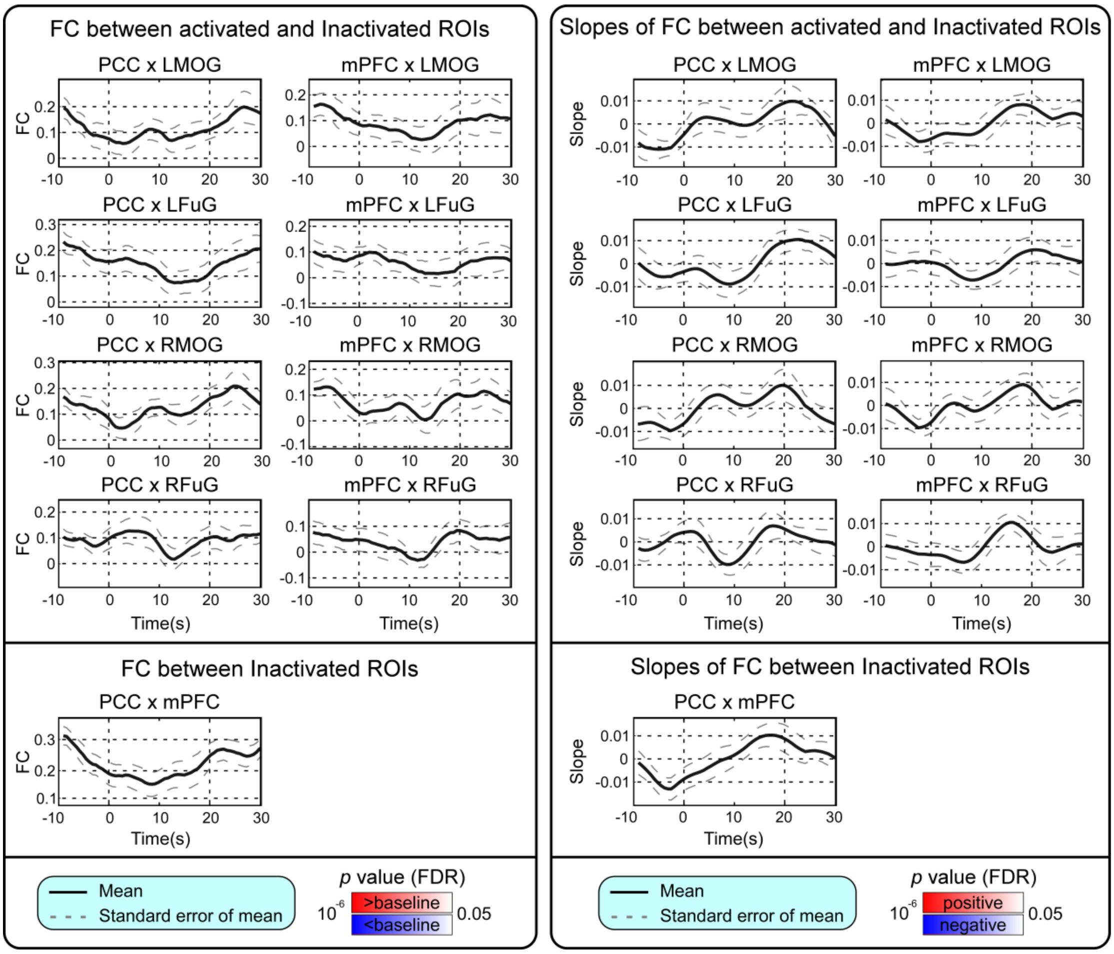

Figure A5 (*corresponding to Figure 4 in the main text, but with a different window size of 14s*). *Left*: Mean and standard error of mean (SEM) of FCs between the activated ROIs and inactivated ROIs, and FC between inactivated ROIs. Two-tailed t-test was used to examine whether the FC after stimulus onset was larger or smaller than that in baseline at each time point. The time points with significantly larger or smaller ($p < 0.05$, FDR corrected) FC than that in baseline were highlighted with red or blue background, respectively. *Right*: Mean and SEM of slopes of FCs between activated ROIs and inactivated ROIs, and slope of FC between inactivated ROIs. Two-tailed t-test was used to examine whether the slope of FC was larger than 0 or smaller than 0 at each time point. The time points with significantly positive or negative ($p < 0.05$, FDR corrected) slope values were highlighted with red or blue background, respectively. Stimulation period was from 0s to 20s. Results were averaged across three blocks and all subjects.

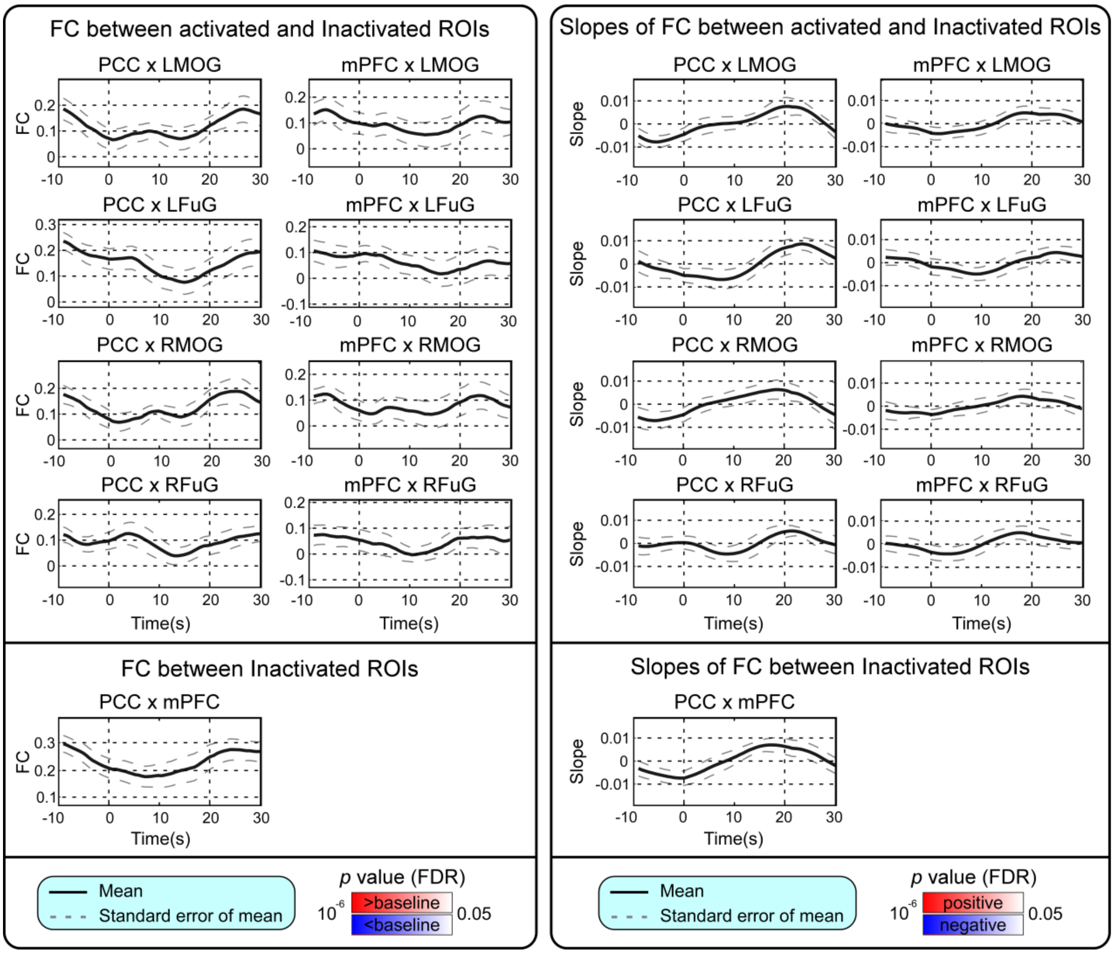

Figure A6 (<u>*corresponding to Figure 4 in the main text, but with a different window size of 18s*</u>). *Left*: Mean and standard error of mean (SEM) of FCs between the activated ROIs and inactivated ROIs, and FC between inactivated ROIs. Two-tailed t-test was used to examine whether the FC after stimulus onset was larger or smaller than that in baseline at each time point. The time points with significantly larger or smaller ($p <$ 0.05, FDR corrected) FC than that in baseline were highlighted with red or blue background, respectively. *Right*: Mean and SEM of slopes of FCs between activated ROIs and inactivated ROIs, and slope of FC between inactivated ROIs. Two-tailed t-test was used to examine whether the slope of FC was larger than 0 or smaller than 0 at each time point. The time points with significantly positive or negative ($p < 0.05$, FDR corrected) slope values were highlighted with red or blue background, respectively. Stimulation period was from 0s to 20s. Results were averaged across three blocks and all subjects.

2. Explanation and Example for regressing out task-related BOLD responses

Regressing out task-related BOLD activities is a crucial step in identifying task-related FC dynamics using sliding-window TVCC estimation. Correlation is a statistical metric that is only applicable for stochastic processes (spontaneous BOLD activity), but not for deterministic processes (task-activated BOLD activity). So, mathematically we need to remove task-related BOLD activities for correct estimation of TVCC. Importantly, if deterministic task-related BOLD activities were not removed before calculating TVCC, they would probably cause dynamic changes of TVCC.

For task-related FC studies, concurrent changes of BOLD signals due to the task stimulation will result in the dynamic changes of TVCC. However, these dynamics are not physiologically relevant, because they are caused by deterministic task-related BOLD activities, which have been well studied. A simple example was shown in Figures A7. Firstly, we generated two random signals (Gaussian processes with mean = 0 and variance = 0.0001; 40 s duration and sampling rate = 40 Hz) without any correlation. Then, one deterministic activation signal (40 s duration and sampling rate = 40 Hz) was generated by convolving an HRF with block designed stimuli (4-Hz stimuli during 0 s to 20 s, where "0 s" denote the stimulation onset). The second deterministic activation signal was generated as the first deterministic activation signal divided by 2. Further, we added the random signals and the deterministic task activations to yield two mixed signals. Finally, we applied sliding window correlation estimation method (using a Gaussian window with window size = 16 s) to calculate the TVCC between random signals as well as mixed signals. As seen from Figures A7, TVCC between the random signals slightly fluctuated around 0 all the time. On the other hand, TVCC between two mixed signals showed largely different dynamic patterns. More precisely, during the concurrent increase and decrease periods (0 s to 10 s and 20 s to 30 s) of task activation, the TVCC between mixed signals exhibited significant transient patterns (increased and then decreased afterward). That is to say, the task-related BOLD activation would bias the exploration of true dynamics on TVCC between spontaneous BOLD activities.

Therefore, it is important to regress out task-related BOLD activities to make sure the correctness of the FC estimation method and to ascertain the physiological significance of observed FC dynamics.

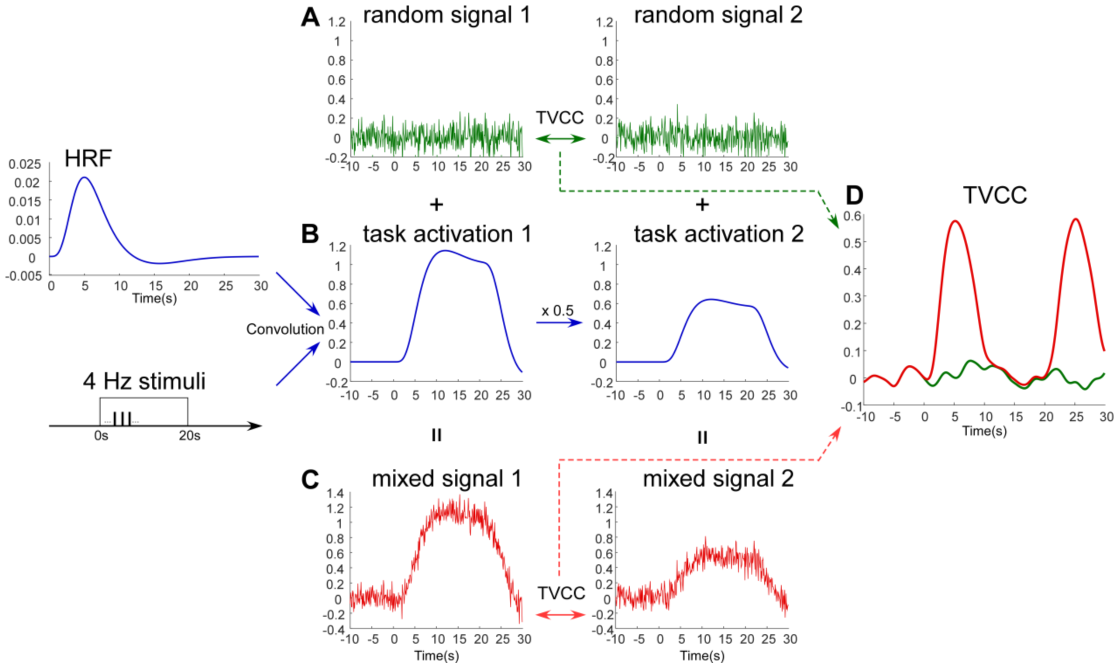

Figure A7. Effect of task-related BOLD activation on estimation of time-varying correlation coefficient (TVCC). A. Two random signals were generated as stochastic processes (Gaussian processes with zero mean and unit variance) without any correlation (correlation = 0). B. Task activations were generated by convolving the HRF with block designed stimuli. C. Mixed signals were obtained by linear combining the random signals and task activations. D. TVCCs were calculated using sliding window correlation estimation method using a Gaussian window with a window size of 16 s (green: TVCC of random signals; red: TVCC of mixed signals).
