# Supplementary figures and images for: Task-related functional connectivity dynamics in a block-designed visual experiment
Source: Front Hum Neurosci. 2015 Sep 30;9:543. doi: 10.3389/fnhum.2015.00543 (PMC4588125; doi:10.3389/fnhum.2015.00543)

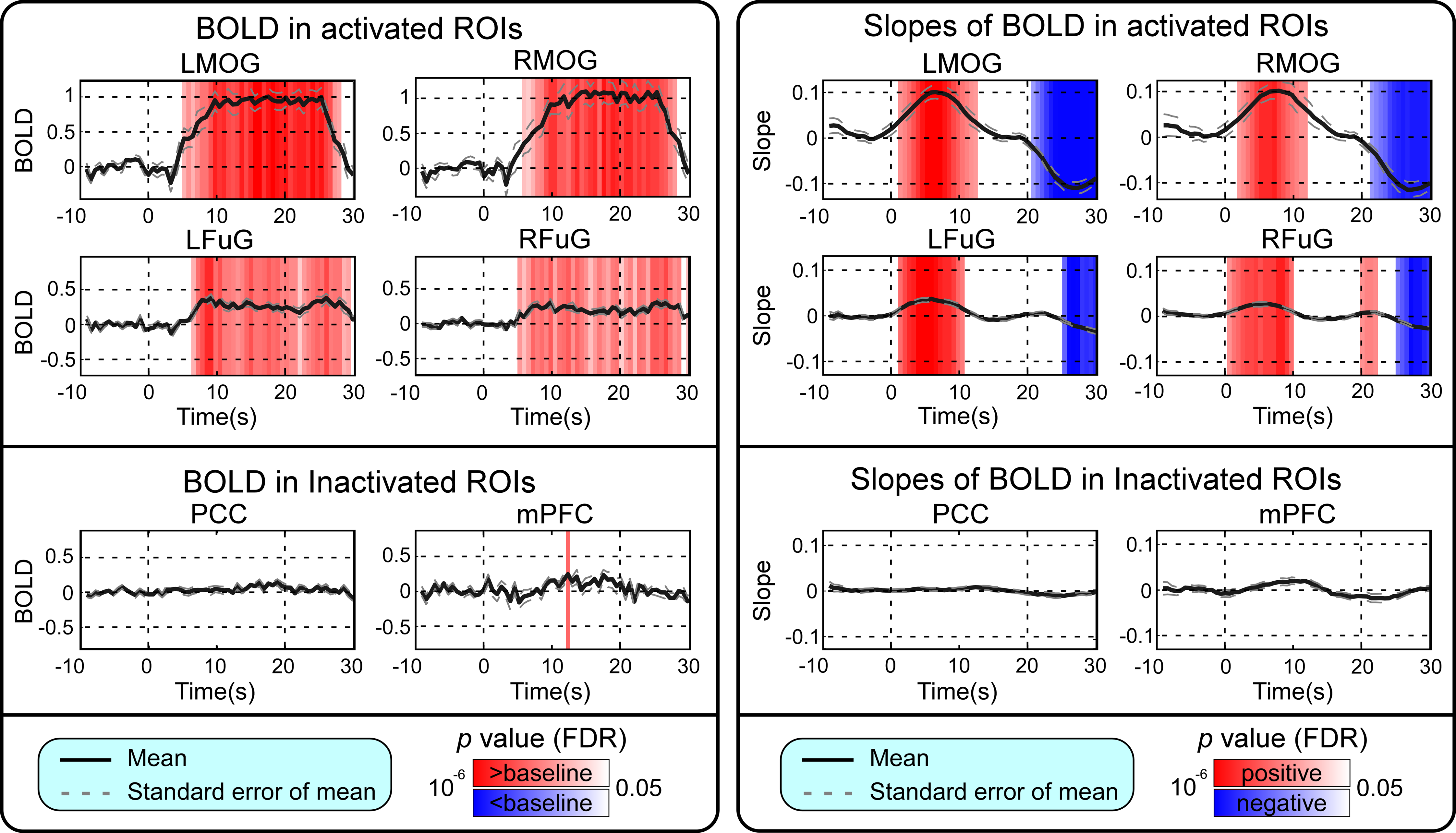

Supplement: Supplementary file 2 [file Image1.TIF]

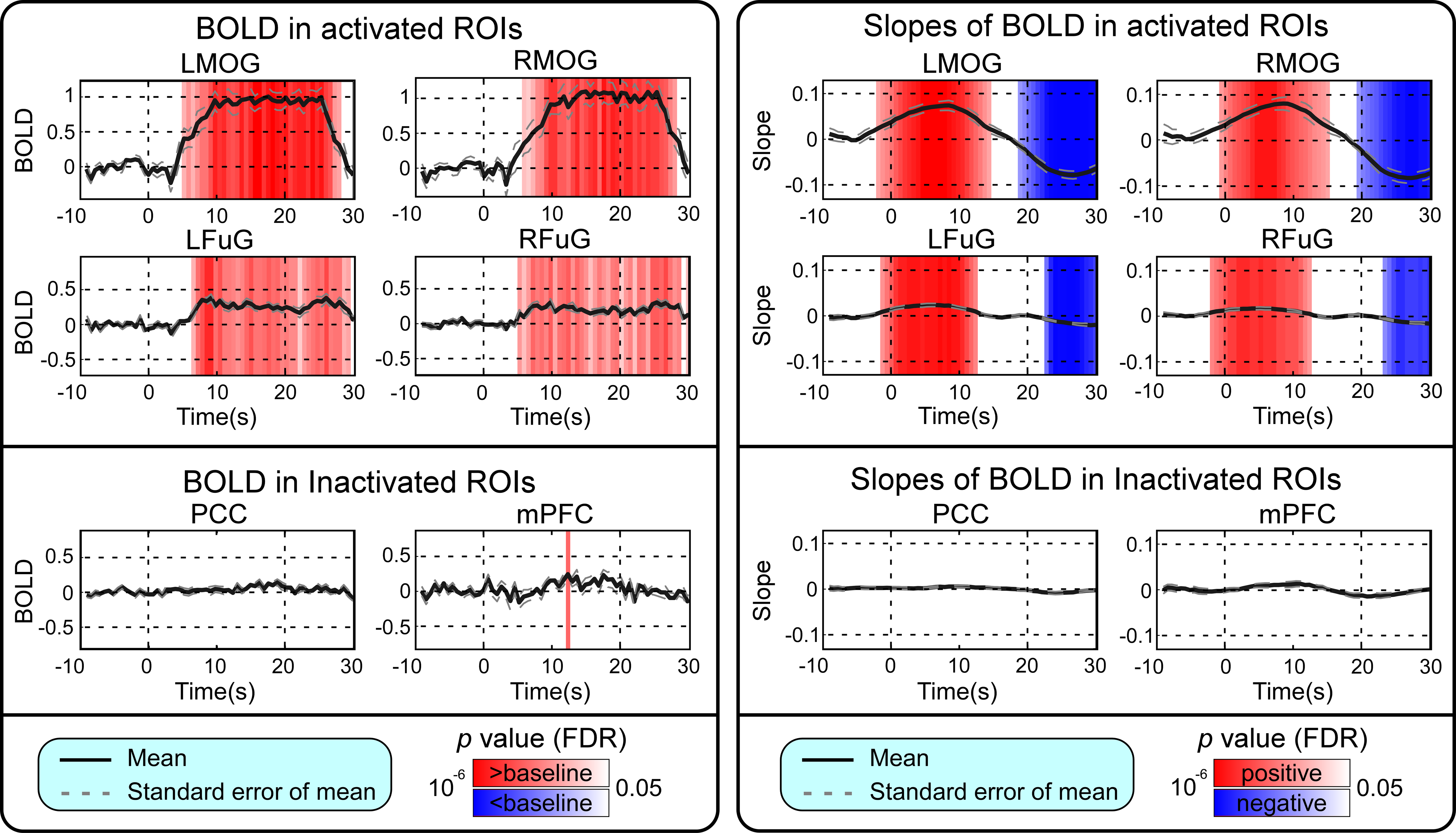

Supplement: Supplementary file 3 [file Image2.TIF]

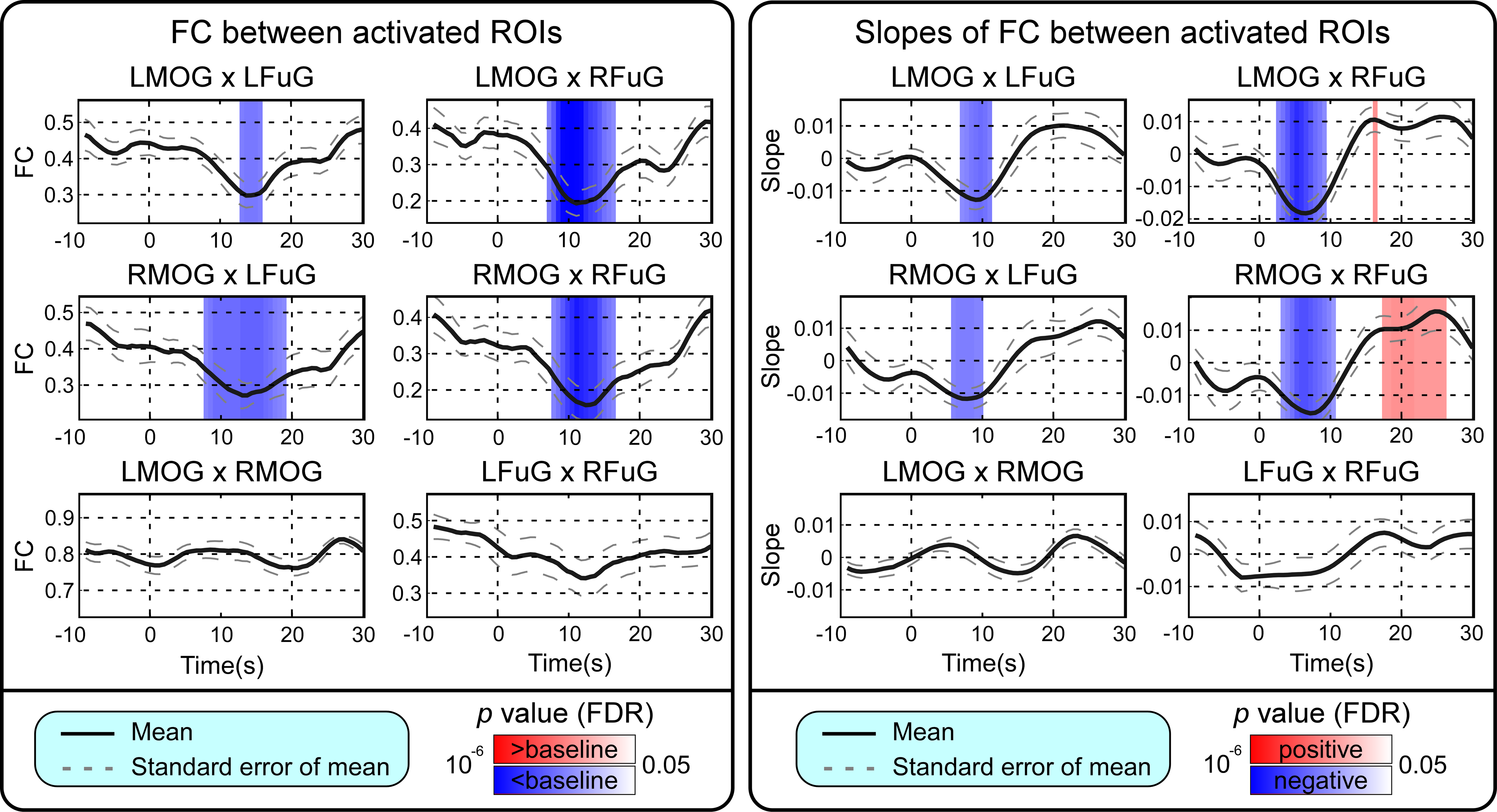

Supplement: Supplementary file 4 [file Image3.TIF]

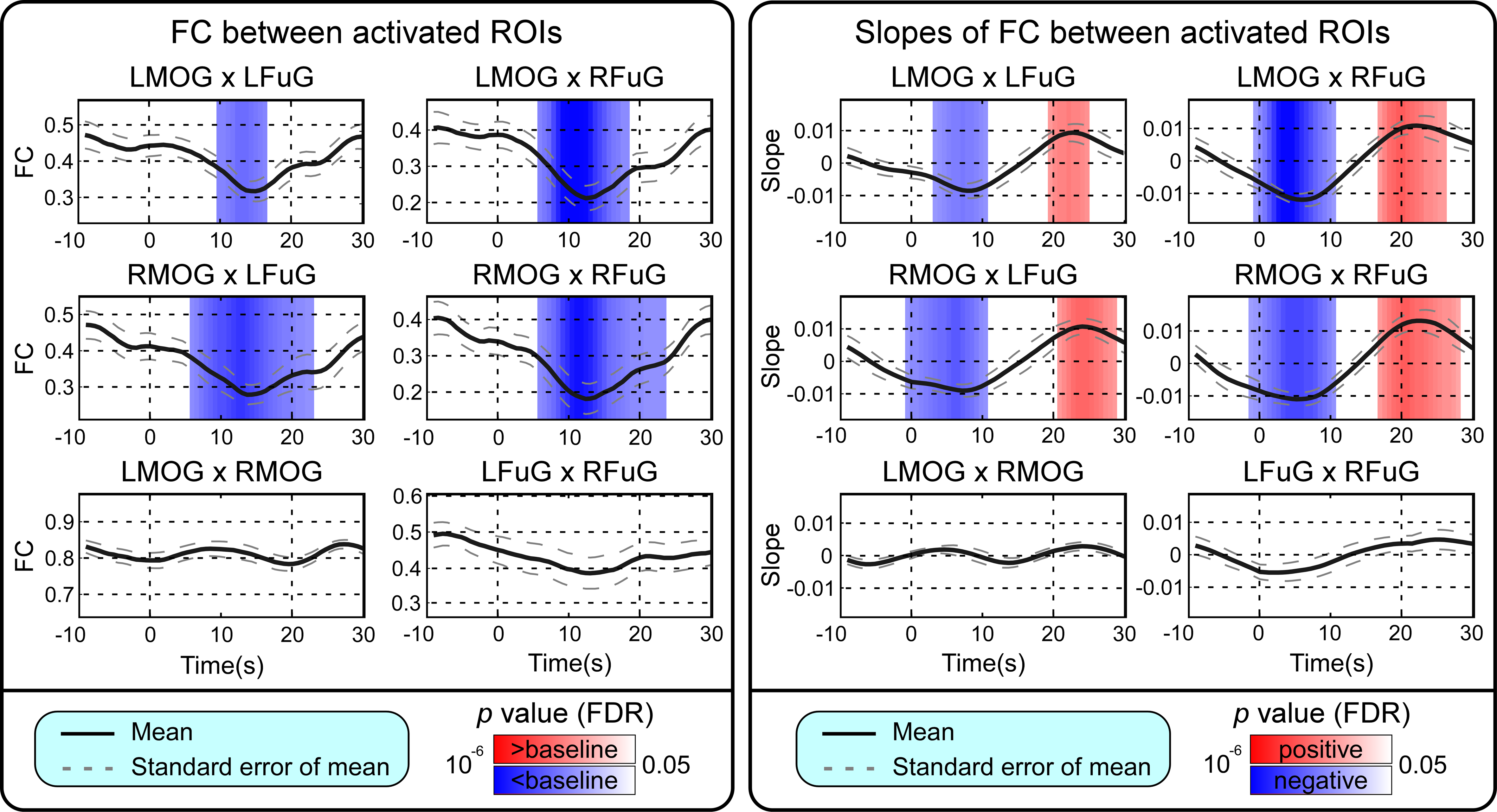

Supplement: Supplementary file 5 [file Image4.TIF]

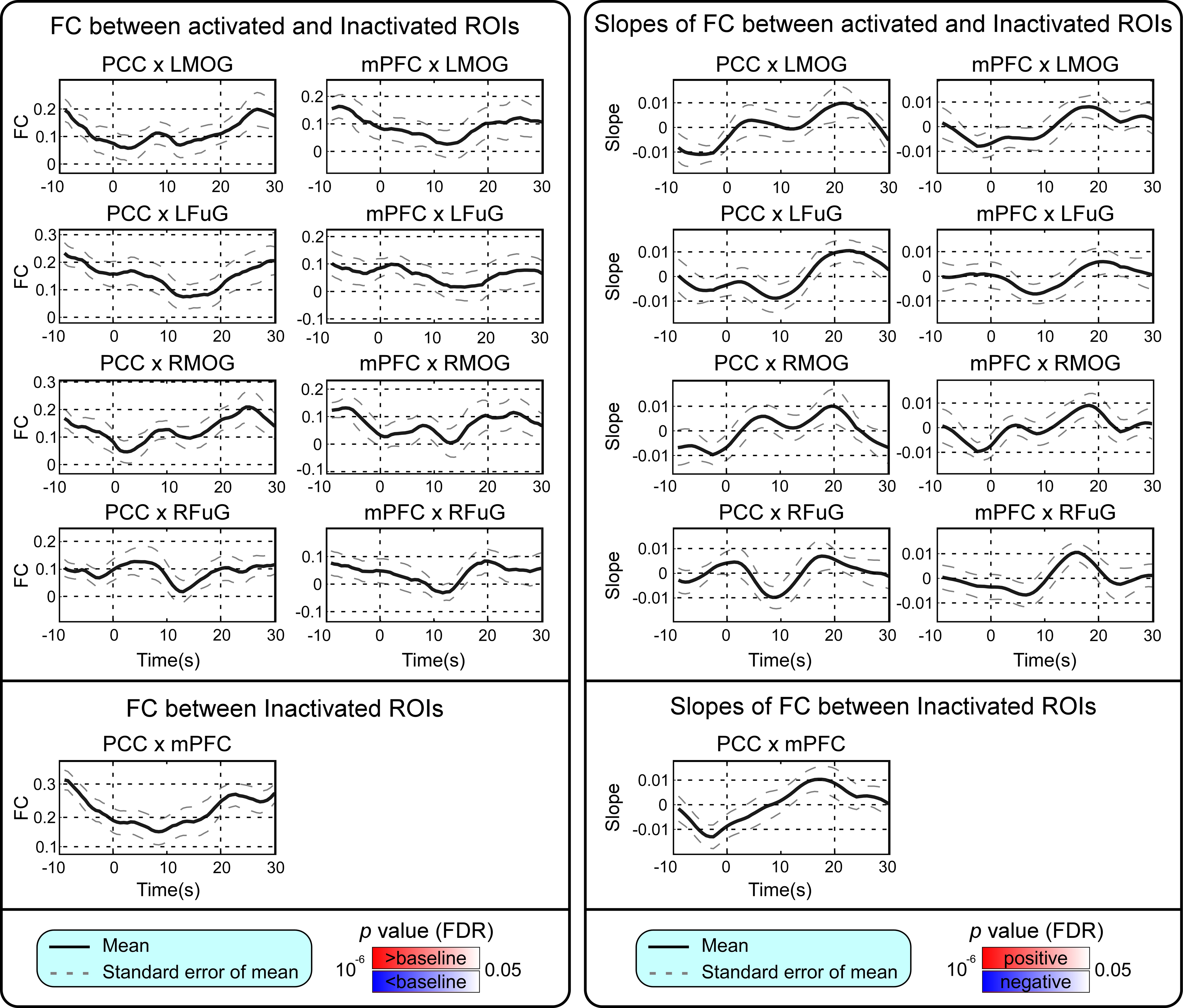

Supplement: Supplementary file 6 [file Image5.TIF]

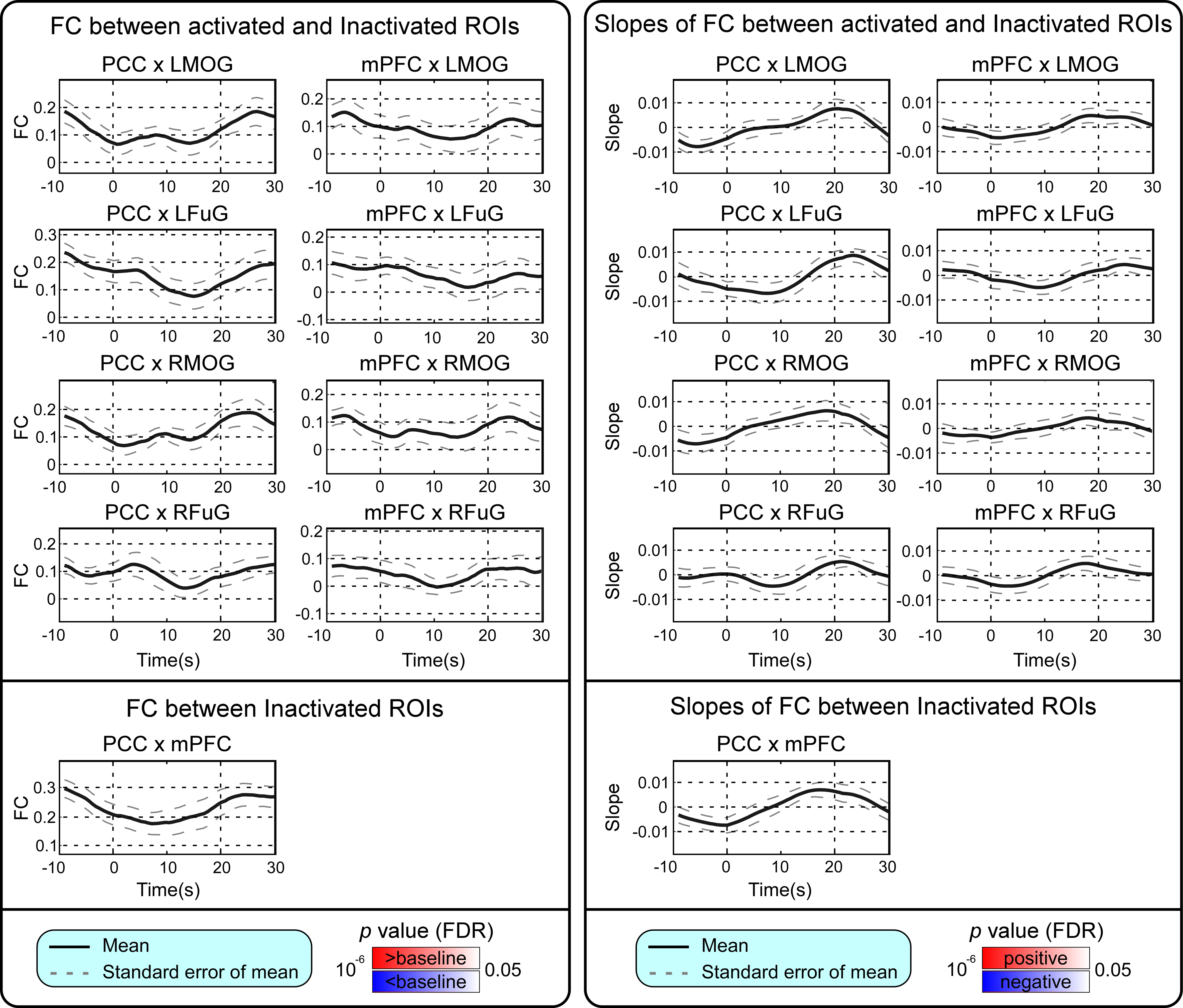

Supplement: Supplementary file 7 [file Image6.TIF]

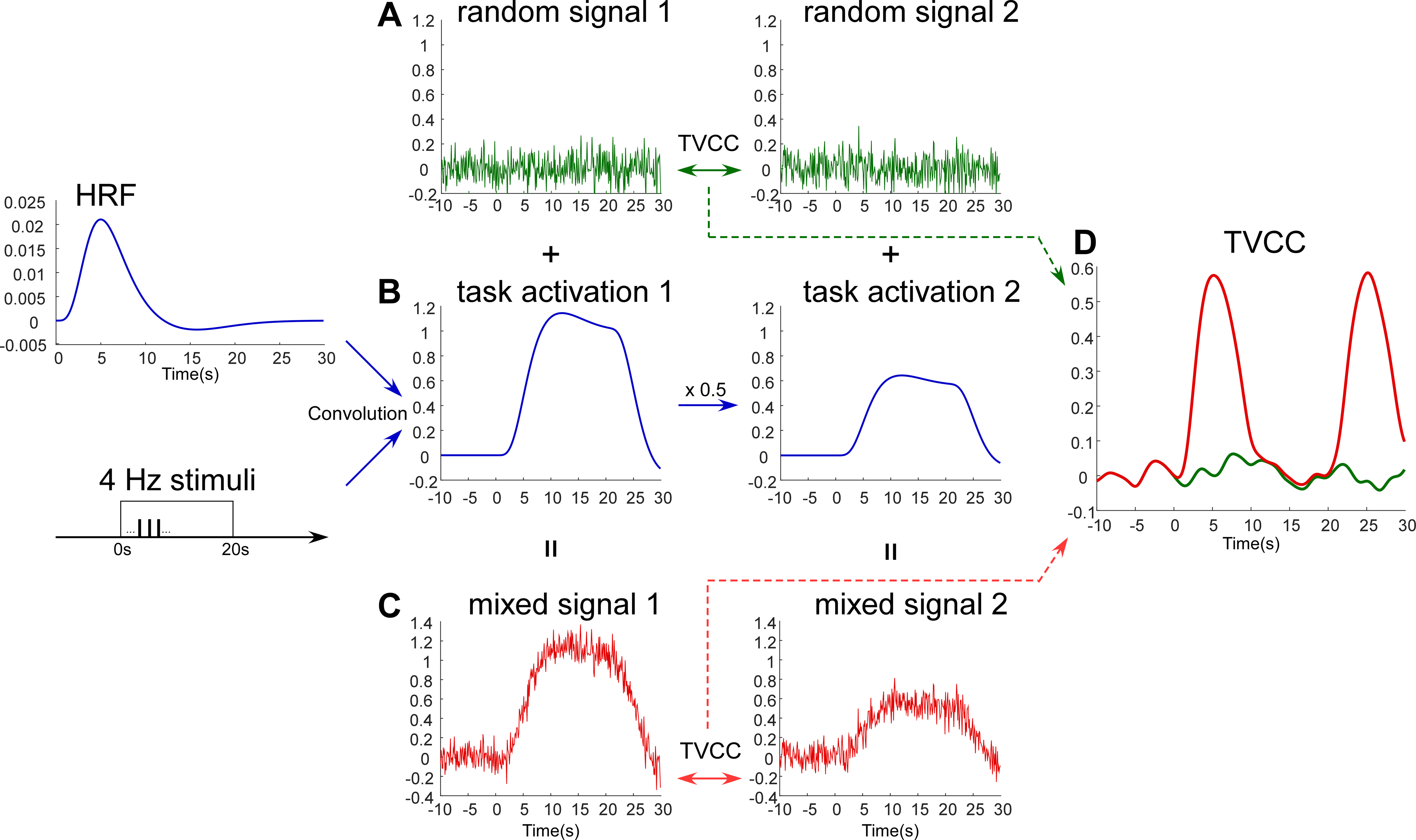

Supplement: Supplementary file 8 [file Image7.TIF]
